# SUMO3 modification by PIAS1 modulates androgen receptor cellular distribution and stability

**DOI:** 10.1186/s12964-019-0457-9

**Published:** 2019-11-21

**Authors:** Nanyang Yang, Sitong Liu, Tian Qin, Xintong Liu, Nobumoto Watanabe, Kevin H. Mayo, Jiang Li, Xiaomeng Li

**Affiliations:** 1 The Key Laboratory of Molecular Epigenetics of MOE, Institute of Genetics and Cytology, Northeast Normal University, 5268 People’s Street, Changchun, Jilin 130024 People’s Republic of China; 2 Hunan Province Cooperative Innovation Center for Molecular Target New Drug Study, Institute of Cytology and Genetics, Hengyang School of Medicine, University of South China, Hengyang, Hunan 421001 People’s Republic of China; 3 College of Life Sciences, Jilin University, Changchun, 130012 People’s Republic of China; 4 Dental Hospital, Jilin University, Changchun, 130021 China; 5 Bioprobe Application Research Unit, RIKEN-Max Planck Joint Research Division, RIKEN Center for Sustainable Resource Science, Wako, Saitama, 351-0198 Japan; 6 Graduate School of Medical & Dental Sciences, Tokyo Medical and Dental University, Tokyo, Japan; 7 Biochemistry, Molecular Biology, and Biophysics, Health Sciences Center, University of Minnesota, Minneapolis, MN 55455 USA

**Keywords:** Sumoylation, PIAS1, SUMO3, Androgen receptor

## Abstract

**Background:**

Abnormal reactivation of androgen receptor (AR) signaling in castration-resistant prostate cancer (CRPC) mainly results from overexpression and down-regulation of AR. Sumoylation of AR can influence its function. However, regulation of AR sumoylation by SUMO E3 ligases PIASs to modify AR distribution and stability are not well understood.

**Methods:**

We assessed the potential effect of SUMO3 modification on AR intracellular localization by immunostaining in AR-negative prostate cancer DU145 cells, and detected the effect of PIAS1/SUMO3 overexpression on AR sumoylation related degradation. Then we characterized AR sumoylation sites involved modified by SUMO3, and the key residue of PIAS1 involved in itself sumoylation and further mediated AR sumoylation (sumo3-conjugated), translocation and degradation. Finally we detected the recognition of PIAS1 (sumoylation ligase) to MDM2, a ubiquin ligase mediated AR degradation.

**Results:**

We demonstrate that SUMO E3 ligase PIAS1, along with SUMO3, mediates AR cytosolic translocation and subsequent degradation via a ubiquitin-proteasome pathway. Although AR sumoylation occurs prior to ubiquitination, the SUMO-acceptor lysine 386 on AR, together with ubiquitin-acceptor lysine 845, contribute to PIAS1/SUMO3-induced AR nuclear export, ubiquitination and subsequent degradation. Moreover, PIAS1 itself is modified by SUMO3 overexpression, and mutation of SUMO-acceptor lysine 117 on PIAS1 can impair AR cytoplasmic distribution, demonstrating the essential role of sumoylated PIAS1 in AR translocation. We further determine that sumoylated PIAS1 interacts with AR lysine 386 and 845 to form a binary complex. Consistent with the effect on AR distribution, SUMO3 modification of PIAS1 is also required for AR ubiquitination and degradation by recruiting ubiquitin E3 ligase MDM2.

**Conclusion:**

Taken together, SUMO3 modification of PIAS1 modulates AR cellular distribution and stability. Our study provided the evidence the crosstalk between AR sumoylation and ubquitination mediated by PIAS1 and SUMO3.

## Background

Androgen receptor (AR) signaling, activated by androgen, plays an essential role in the initiation and progression of prostate cancer (PCa) [1, 2]. Despite the initial clinical benefit from androgen deprivation therapy, most patients eventually relapse with a more aggressive castration-resistant PCa (CRPC) with no curative therapy [3]. In CRPC, AR signaling abnormally activates even at low androgen levels post-castration [4], and occurs via several mechanisms, including AR gene amplification and overexpression [5], abnormal AR stability regulation [6], AR mutations or splice variant [7, 8], altered expression of AR co-factors [9], or altered interactions between AR and co-factors, etc. AR is overexpressed in up to 80% of CRPC patient samples [6, 10, 11] and it is the only consistently up-regulated gene in all resistant xenograft models [12], suggesting that the AR gene overexpression or the increased AR protein stability is the primary underlying mechanism involved in AR reactivation in CRPC [6]. Thus, down-regulation of AR protein level by increasing AR degradation pathway may present a good strategy to controlling PCa in patients with CRPC.

Post-translational protein modifications, such as ubiquitination or sumoylation, can regulate protein stability and affect protein levels in cells. Poly-ubiquitination of proteins with a K48-linked ubiquitin chain usually targets protein degradation via the 26S proteasome [13, 14]. Similar to other nuclear receptors, AR is subject to regulation by the ubiquitin-proteasome pathway [13], and some proteins, such as MDM2 or ChIP, can function as ubiquitin E3 ligases to ubiquinate AR [14–16]. The process of enzyme-mediated, small ubiquitin-related modifier (SUMO) protein conjugation is termed sumoylation. The SUMO conjugation cascade consists of the SUMO E1 SAE1/2 heterodimer, SUMO E2 Ubc9, and a restricted set of E3 enzymes comprising PIAS family members. Four SUMO analogues designated SUMO1, and 2/3, are typically expressed in vertebrates. SUMO2 and 3 are ~ 96% identical, whereas SUMO1 has only ~ 45% identity with both SUMO2 and 3 [17]. SUMO modification can regulate e.g. protein-protein or protein-DNA interactions, protein subcellular translocation, sub-nuclear structure formation, and protein stability [14, 18, 19].

AR is a substrate for sumoylation, and PIAS family proteins act as E3 ligases to promote AR sumoylation [13]. SUMO1 modification promoted by PIAS1 and PIASxα, appears to reduce the transcriptional activity of AR in presence of SUMO1 [20], without affecting its sub-nuclear localization [21] and DNA-binding capability [22]. Different from the negative effect of SUMO-1 conjugation on AR-initiated transcription, SUMO3 is supposed to either inhibit or stimulate AR transactivation, depending on the type of cell lines. In addition, PIAS1 and PIASxα enhance the AR-dependent transcription in the absence of sumoylation [23]. Although these studies implicate SUMO3 and PIASs in regulation of AR mediated transactivation, Here, we the potential effects of common SUMO E3 ligases PIASs and their catalyzing SUMO3 modification on AR cellular distribution and stability are still unclear.

In this study, we discovered that AR is exported from the nucleus and degraded by PIAS1 together with SUMO3. Although increased sumoylation levels of AR are detected, only mutation of AR sumoylation site K386, but not K520, prevents cytoplasmic translocation and degradation of AR. This suggests that sumoylation site K386 plays a crucial role in nuclear export and subsequent degradation in a sumoylation-independent manner. PIAS1 itself, as a SUMO E3 ligase, is also modified by SUMO3, which results in cytoplasmic translocation of AR. Specific recruitment of the mouse homologue of double minute 2 protein (MDM2) ubiquitin E3 ligase of AR participates in the regulation of AR turnover. These findings reveal a novel role for self-sumoylation of SUMO E3 ligase PIAS1 in the regulation of AR cellular distribution and degradation, and also unveils a previously unknown crosstalk between components of the sumoylation (SUMO3 modified PIAS1) and ubiquitination machinery (MDM2) in the degradation of the AR protein.

## Results

### PIAS1/SUMO3 overexpression promotes cytoplasmic translocation of AR from nucleus

**Fig. 1:**
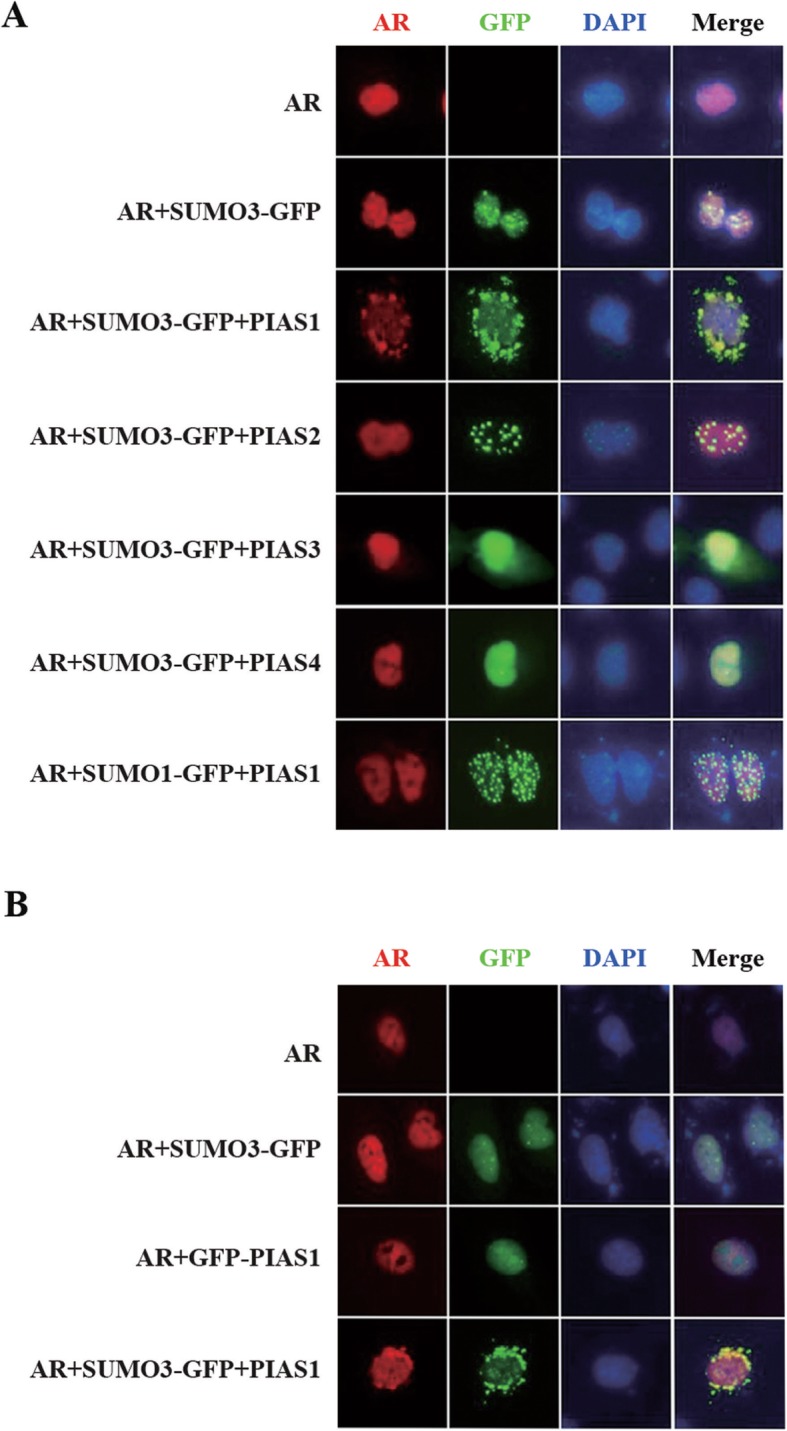
Subcellular localization of AR in the presence of PIAS and SUMO. (**a/b**) DU145 cells in a 12-well plate were cotransfected with indicated plasmids for 48 h. Cells were then fixed and stained with anti-AR rabbit polyclonal antibody, then PI-conjugated anti-rabbit IgG antibody (red). Nuclei were visualized by DAPI staining. Representative images of transfected cells were acquired using immunofluorescence microscope

Sumoylation exerts diverse effects, ranging from regulation of transcription to intracellular trafficking [14]. To assess the potential effect of SUMO3 modification on AR intracellular localization, we immunostained exogenous AR when co-expressed with PIAS family and SUMO3 in AR-negative prostate cancer DU145 cells. As shown in Fig. 1a, after 48 h transfection, AR itself or AR co-transfected with SUMO3, was predominantly found in the nucleus. Furthermore, co-expression of PIAS2, PIAS3, or PIAS4 with AR did not influence AR nuclear localization in the presence of GFP-SUMO3. However, co-transfection of PIAS1 and SUMO3 with AR shifted both partial AR and SUMO3 from the nucleus to the cytoplasm in the perinuclear space. This specific cytoplasmic distribution of ectogenous AR could not be detected in cells when PIAS1 and GFP-SUMO3 were separately co-transfected (Fig. 1b). Collectively, these findings illustrate that PIAS1, together with SUMO3, promotes AR export from the nucleus.

### PIAS1/SUMO3 overexpression induces the degradation of ectopic AR via a proteasome-dependent pathway

**Fig. 2:**
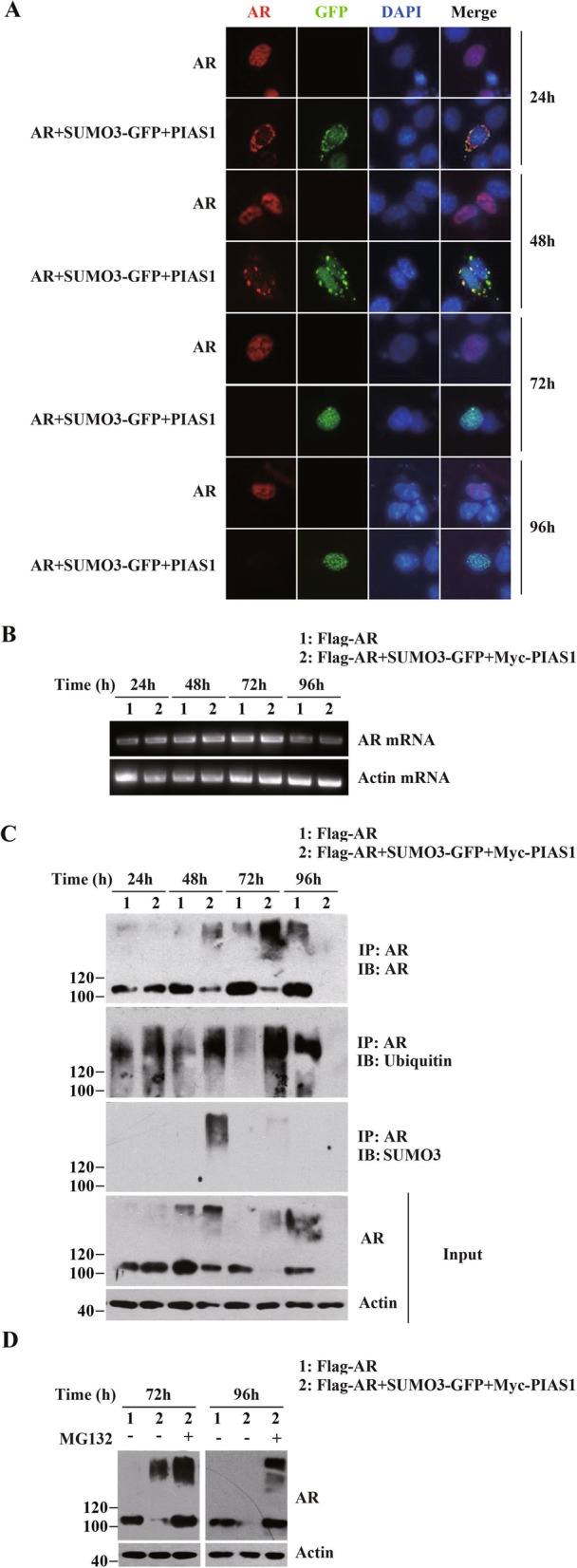
PIAS1 together with SUMO3 facilitates ubiquitin-proteasome mediated AR degradation. (**a**) DU145 cells in a 12-well plate were transiently transfected with empty vector, AR or AR along with PIAS1 and GFP-SUMO3. Cells were then fixed at different transfection periods (24 h, 48 h, 72 h and 96 h), and stained for AR (red). Representative images of transfected cells were shown. (**b**) DU145 cells were transfected with plasmids as described in A. The AR mRNA or beta-actin mRNA levels were analyzed by reverse-transcriptional PCR at indicated time course after transfection (24 h, 48 h, 72 h and 96 h). (**c**) DU145 cells were transfected with plasmids as described in A. Whole cell lysates at indicated time course after transfection were generated together and immunoprepapited with anti-AR antibody. The Immunoprecipitate was detected by anti-AR (IP, top panel), anti-ubiquitin (IB, second panel), and anti-SUMO3 immunoblotting (IB, third panel). Whole-cell lysates (Input) were immunoblotted with anti-AR (fourth panel) or anti- actin (bottom panel) antibodies. (**d**) DU145 cells were cotransfected with empty vectors, AR or AR together with GFP-SUMO3 and PIAS1. Cells were then treated with or without MG132 (5 μM) for 16 h before cells were collected at 72 h or 96 h as indicated after transfection. Whole-cell lysates were immunoblotted with anti-AR or anti-actin antibodies

Because of the observation that partial AR translocation into the cytoplasm occured at 48 h post-transfection with PIAS1 and SUMO3, we followed the kinetics of localization and immunofluorescence intensity changes of AR induced by PIAS1 and SUMO3 at different time points (i.e. 24 h, 48 h, 72 h and 96 h, Fig. 2a). Here, we found that the cytoplasmic distribution of ectogenous AR was also detected at 24 h post-transfection. However, AR staining was either much weaker or not detected at longer transfection periods (72 h and 96 h), suggesting down-regulation of AR by PIAS1 and SUMO3 following its translocation into the cytoplasm. Next, we detected the gene transcriptional levels of AR mRNA induced by PIAS1 and SUMO3 at different time points (i.e. 24 h, 48 h, 72 h and 96 h), and found that mRNA levels did not change (Fig. 2b).

Some proteins, included AR, have been reported translocated to the cytoplasm for degradation with an aggregation pattern [24, 25]. We followed the kinetics of protein levels of AR induced by PIAS1 and SUMO3 at different time points (i.e. 24 h, 48 h, 72 h and 96 h, Fig. 2c) by Western blot analysis, and found that AR protein levels were apparent reduced at 72 h and 96 h (Fig. 2c). Next, we examined whether PIAS1 and SUMO3 together affected AR ubiquitination, by using anti-Ubiquitin antibody in co-immunoprecipitation analyses, determined for 72 h dramatically upregulated ubiquitination of AR (Fig. 2c), demonstrating that AR levels were depleted by protein degradation. Further, the degradation of ectopic AR in cells co-expressing PIAS1 and SUMO3 was prevented by treatment with proteasome inhibitor MG132 (Fig. 2d). These findings indicate that PIAS1/SUMO3 co-overexpression induce proteolytic degradation of AR mediated via the ubiquitination-proteasome dependent protein degradation pathway.

### Disruption of sumoylation site K386 or ubiquitination site K845 on AR abrogates PIAS1/SUMO3 co-expression-induced AR cytoplasmic translocation and subsequent ubiquitination-mediated degradation

PIAS1 and other PIAS family members are SUMO-E3 ligases that facilitate SUMO conjugation to specific substrates [14, 21]. With immunoprecipitation assays in AR/PIAS1/SUMO3 co-transfected cells, we observed that there are additional AR-reactive, slowly migrating bands at 48 h before AR was ubiquitilated at 72 h (Fig. 2c IP:AR). By SUMO3 immunobloting, we found that these additional AR-reactive slowly migrating bands were AR-sumolated high-molecular weight species (Fig. 2c IB: SUMO3). This indicates that in cells expressing PIAS1 together with SUMO3, a fraction of ectopic AR undergoes SUMO3 modification.

**Fig. 3:**
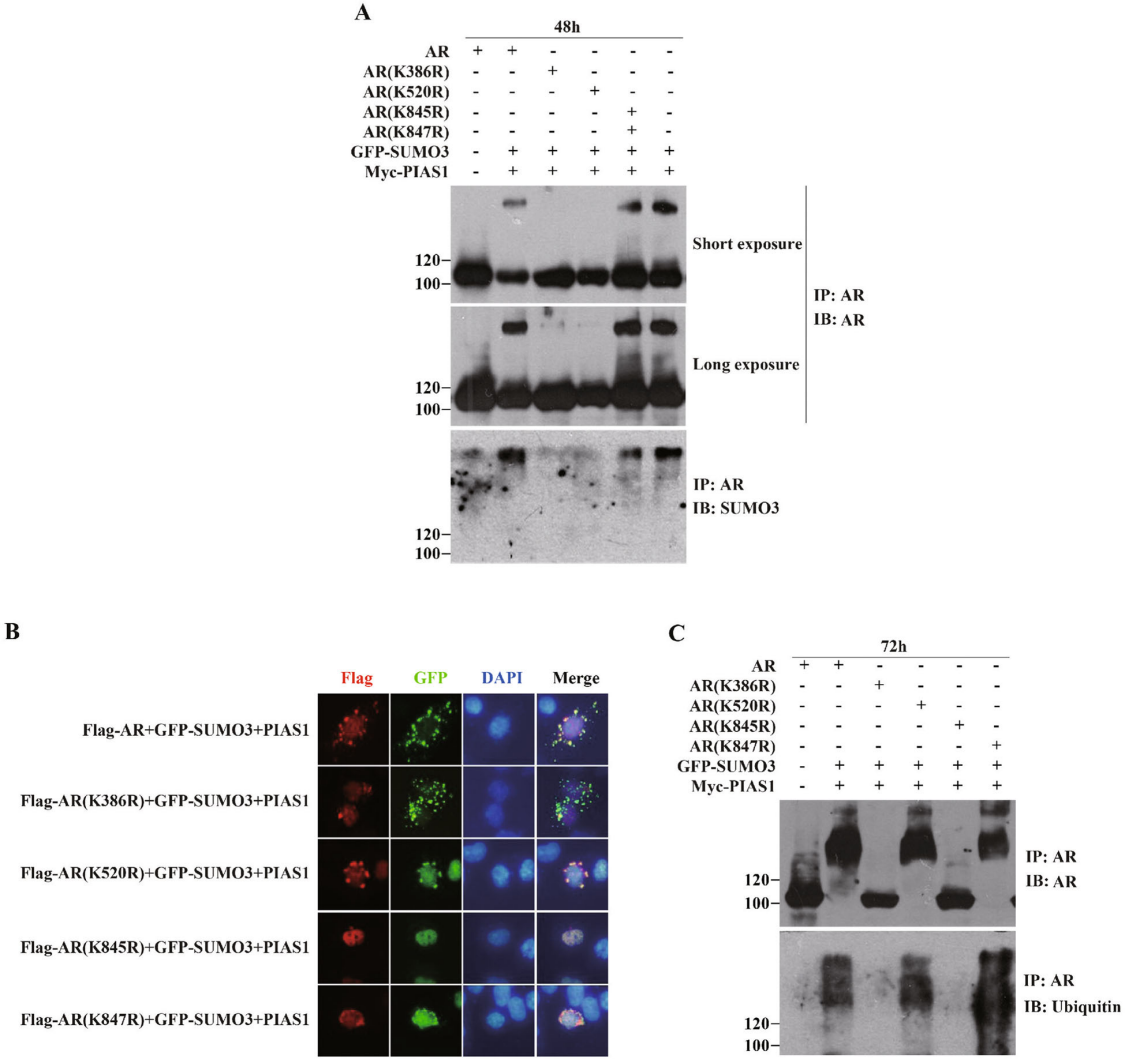
Lysines 386 and 845 of AR are critical for PIAS1/SUMO3 overexpression induced nuclear export and degradation of AR. (**a**) DU145 cells cotransfected with myc-PIAS1, GFP-SUMO3 and wildtype AR or various point mutants of AR expression constructs as indicated for 48 h were subjected to immunoprecipitation with anti-AR antibody (IP), and this was followed by immunoblotting with anti-AR (IP) or anti-SUMO3 (IB) antibodies. (**b**) DU145 cells cotransfected with plasmids as described in A were fixed and stained with anti-flag mouse monoclonal antibody, then PI-conjugated anti-mouse IgG antibody (red). Nuclei were visualized by DAPI staining. Representative images of transfected cells were acquired using immunofluorescence microscope. (**c**) DU145 cells were cotransfected with plasmids as described in A for 72 h and then subjected to immunoprecipitation with anti-AR antibody, and followed by immunoblotting with anti-AR (IP) or anti-ubiquitin (IB) antibodies

We next investigated whether SUMO3 modification of AR contributes to its nuclear export promoted by PIAS1 and SUMO3. Two reported AR sumoylation site mutants: K386R and K520R [13] and AR ubiquitination site mutants AR K845R and AR K847R [13] were used. As shown in Fig. 3a, whereas AR K520R and AR K847R were present in the cytoplasm in PIAS1 and SUMO3 co-transfected cells, AR K386R or K845R markedly impaired cytoplasmic localization of AR. Since mutations of K386 or K520 both significantly reduced PIAS1 and SUMO3 promoted AR SUMO3 modification (Fig. 3b), we concluded that sumoylation site K386, but not K520, was essential for PIAS1 and SUMO3 induction of AR cytoplasmic translocation.

Recent emerging data suggest that sumoylation can also target a protein for ubiquitin-proteasomal-mediated degradation, in addition to its competition function with ubiquitination [26, 27]. To explore the relationship between SUMO3 modification and ubiquitination-proteasomal degradation of AR, DU145 cells were separately transfected with AR or mutants in the presence of PIAS1 and SUMO3. Immunoblot analysis of AR immunoprecipitate in these transfected cells with AR or ubiquitin antibody were performed. Consistent with our previous results (Fig. 2c), PIAS1 together with SUMO3 enhanced ubiquitination of wild type AR and further reduced AR protein levels. In addition, enhanced ubiquitination and decreased protein levels were also observed in sumoylation site mutant AR K520R and ubiquitination site mutant AR K847R. However, the other two mutants, AR K386R and AR K845R, showed no effect of PIAS1 and SUMO3 on ubiquitination and protein expression levels (Fig. 3c). These data demonstrated that AR sumoylation site K386 and ubiquitination site K845, but not SUMO3 modification, were necessary for PIAS1/SUMO3 co-expression-induced AR cytoplasmic translocation and subsequent ubiquitination-mediated degradation.

### Modification of PIAS1 itself by SUMO3 at 117th lysine residue

**Fig. 4:**
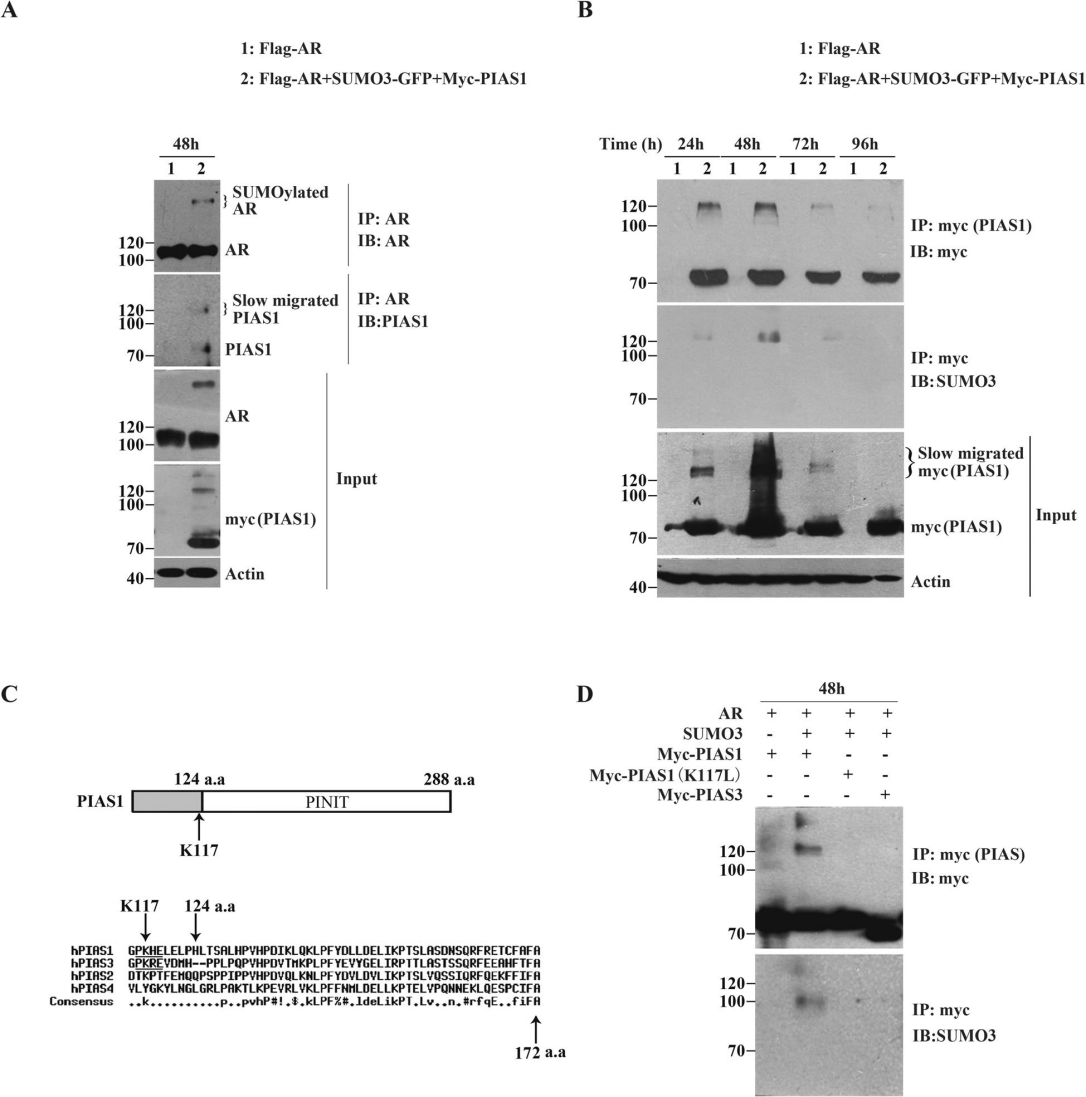
PIAS1 itself was modified by SUMO3 at 117th lysine residue. (**a**) DU145 cells were cotransfected with plasmids as indicated for 48 h. Whole-cell lysates were immunoprepapited with anti-AR antibody and then analyzed by immunoblot analysis using the indicated antibodies against AR (IP, top panel) and PIAS1 (IB, second panel). Whole-cell lysates (Input) were also immunoblotted with anti-AR (third panel), anti-PIAS1 (fourth panel) or anti-actin (bottom panel) antibodies. (**b**) DU145 cells were co-transfected with plasmids as indicated for different transfection periods (24 h, 48 h, 72 h and 96 h). Whole cell lysates generated together were immunoprepapited with anti-myc antibody. The myc-immunoprepapite was then detected by anti-myc (IP, top panel) and anti-SUMO3 immunoblotting (IB, second panel). Whole-cell lysates (Input) were also immunoblotted with anti-myc (third panel) or anti-actin (bottom panel) antibodies. (**c**) Diagrammatic representation of putative typical sumoylation site on human PIAS1 sequense predicted by using the GPS-SUMO software. Analysis of human PIAS1 sequence indicated the presence of only one putative typical sumoylation site, Lys-117, which located near to the PINIT domain of PIAS1. (**d**) DU145 cells were co-transfected with plasmids as indicated for 48 h and Whole cell lysates were immunoprepapited with anti-myc antibody. The myc-immunoprepapite was then detected by anti-myc (IP, top panel) and anti-SUMO3 immunoblotting (IB, second panel)

PIAS proteins were reported can be self-sumoylated in vitro, as well as when both PIAS and SUMO are overexpressed in cells [21, 28–30]. In cells expressing PIAS1 together with SUMO3 in the presence of AR, we often observe that there are one or two additional at all transfection time points (Fig. 4b Input). Moreover, in the AR-immunoprecipitate of these transfected cells, we have detected a discernible PIAS1 band having a high molecular weight ~ 120 kDa, in addition to PIAS1 having a normal molecular weight ~ 71 kDa (Fig. 4a). To test whether PIAS1 is sumoylated by ectopic SUMO3 that also regulates AR, the lysates of cells expressing myc-PIAS1 together with SUMO3 and AR, were immunoprecipitated with anti-myc tag antibody (Fig. 4b IP:myc), and further the anti-SUMO3 antibody, detected the same slowly migrating bands (Fig. 4b IB:SUMO3). These results suggest the possibility of SUMO3 modification of PIAS1 itself.

SUMO modification of target proteins occurs via lysines that exist in aψKXD/E consensus sequence [31]. Since no report on the exact sumoylation site in PIAS1, we first searched for potential sumoylation sites by bioinformatic analysis [32]. Using GPS-SUMO software, we found only one potential typical consensus sequence for sumoylation, PK117HE, in PIAS1, located close to its PINIT domain (Fig. 4c). PIAS1 acting as a common SUMO-E3 ligase can promote sumoylation of many proteins, including AR. To confirm the sumoylation site in PIAS1, we mutated K117 to leucine and compared the SUMO3 modification levels with wild type PIAS1 in presence of SUMO3 and AR. As shown in Fig. 4d, the slowly migrating band of myc-PIAS1 (which had been detected in cells expressing myc-PIAS1, SUMO3 and AR) disappeared when K117 was mutated (Fig. 4d IP:myc lane 2 and 3). Furthermore, the high-molecular-weight band at ~ 120 kDa was absent in the myc-PIAS1 K117 L immunoprecipitate (Fig. 4d IB:SUMO3 lane 2 and 3). These results suggest that PIAS1 itself is SUMO3 modified in the presence of SUMO3 and AR, and that K117 is the sumoylation site in PlAS1.

### Formation of SUMO3-modified PIAS1 and AR complexes via AR K386 sumoylation and AR K845 ubiquitination

**Fig. 5:**
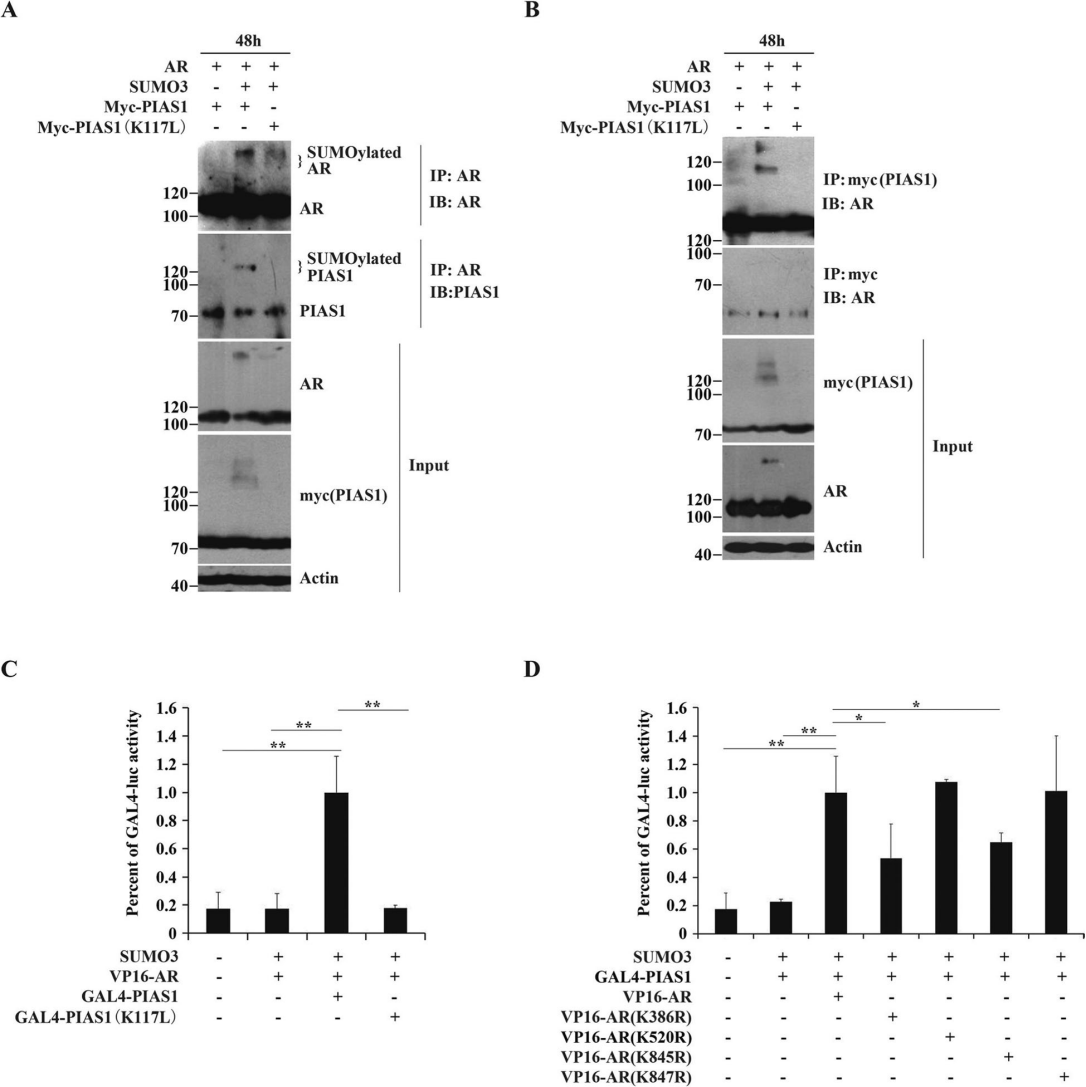
The formation of AR and sumoylated PIAS1 complex. (**a/b**) DU145 cells were cotransfected with plasmids as indicated for 48 h and the total amount of plasmids per well was normalized by empty vectors. Whole-cell lysates were immunoprepapited with anti-AR or anti-myc antibodies. The immunoprepapite was then detected by indicated antibodies against AR (IP, top panel of **a**) and anti-PIAS1 (IB, second panel of **a**), or against myc (IP, top panel of **b**) and anti-SUMO3 (IB, second panel of **b**). Whole-cell lysates (Input) were also immunoblotted with anti-AR (third panel of **a**), anti-myc (fourth panel of **a**) and anti-actin (bottom panel of **a**) antibodies, or with anti-myc (third panel of **b**), anti-AR (fourth panel of **b**) and anti-actin (bottom panel of **b**) antibodies. (**c**) The mammalian two-hybrid assay was performed in DU145 cells. Cells were transiently transfected in 48-well with 100 ng 5 × GAL4-luc, 25 ng Renilla luciferase reporter,30 ng SUMO3, 30 ng VP16-AR and 30 ng GAL4-PIAS1 or GAL4-PIAS1 (K117 L) as indicated. The total amount of plasmids per well was normalized in all transfections by the addition of empty vectors. Transfected cells were grown for 48 h and then harvested for luciferase assay. Values represent mean ± S.D. *P < 0.01. (**d**) DU145 were transiently transfected in 48-well with 100 ng 5 × GAL4-luc, 25 ng Renilla luciferase reporter,30 ng SUMO3, 30 ng GAL4-PIAS1, and 30 ng VP16-AR or various points mutants of VP16-AR constructs as indicated for 48 h, and then harvested for luciferase assay. Values represent mean ± S.D. *P < 0.05,**P < 0.01

To examine whether SUMO3-modified PIAS1 directly interacts with AR, we compared the binding of wild type PIAS1 and mutant K117 L to AR in present of SUMO3. Immunoprecipitation with anti-AR antibodies showed that in cells expressing AR and PIAS1 as a control, co-precipitation between AR and PIAS1 occurred (Fig. 5a lane 1 IB: PIAS1). Notably, other than PIAS1(~71KD), the slow migrating PIAS1 (~120KD) that represents sumoylated PIAS1, was in the AR-immunoprecipitate prepared from cells co-expressed with AR, PIAS1 and SUMO3, but not from cells co-transfected with AR, SUMO3 and PIAS1 K117 L. Reverse co-immunoprecipitation using the myc antibody (Fig. 5b) and only AR, showed interactions with both PIAS1 and its SUMO3-modified analog. These data suggest that the novel SUMO3-modified PIAS1/AR complex formed in addition to the PIAS1/AR complex. The SUMO3-PIAS1/AR complex was confirmed by mammalian two-hybrid assays (Fig. 5c). As expected, the markedly elevated reporter activity that resulted from co-transfection of GAL4-PIAS1 with VP-16 in the presence of SUMO3, was partially reversed when GAL4-PIAS1 was substituted with GAL4-PIAS1 K117 L.

Because sumoylation site K386 and ubiquitination site K845, but not sumoylation of AR, are essential for PIAS1/SUMO3-induced AR cytoplasmic translocation and subsequent degradation, we investigated whether these two AR sites are required binding to SUMO3-modified PIAS1. As shown in Fig. 5d, apparent activation of the 5 × GAL4-luc reporter was observed when cells were co-transfected with VP16-AR and GAL4-PIAS1 in the presence of SUMO3. This indicates the presence of strong AR/ PIAS1- or SUMO3-modified PIAS1 interactions. Moreover, AR mutants K386R or K845R displayed partial loss of PIAS1- or SUMO3-modified PIAS1 interactions, whereas AR mutants K520R or K847R had no effect on AR-PIAS1- or SUMO3-modified PIAS1 interactions. Since both K386R and K845R mutants did not abolish the AR/PIAS1 interaction in the absence of SUMO3 (data not shown), our findings led us to conclude that K386 and K845 contribute to interactions between AR and SUMO3-modified PIAS1.

### SUMO3 modification of PlAS1 K117 is necessary for PIAS1/SUMO3 over-expression-mediated AR cytoplasmic translocation and degradation

**Fig. 6:**
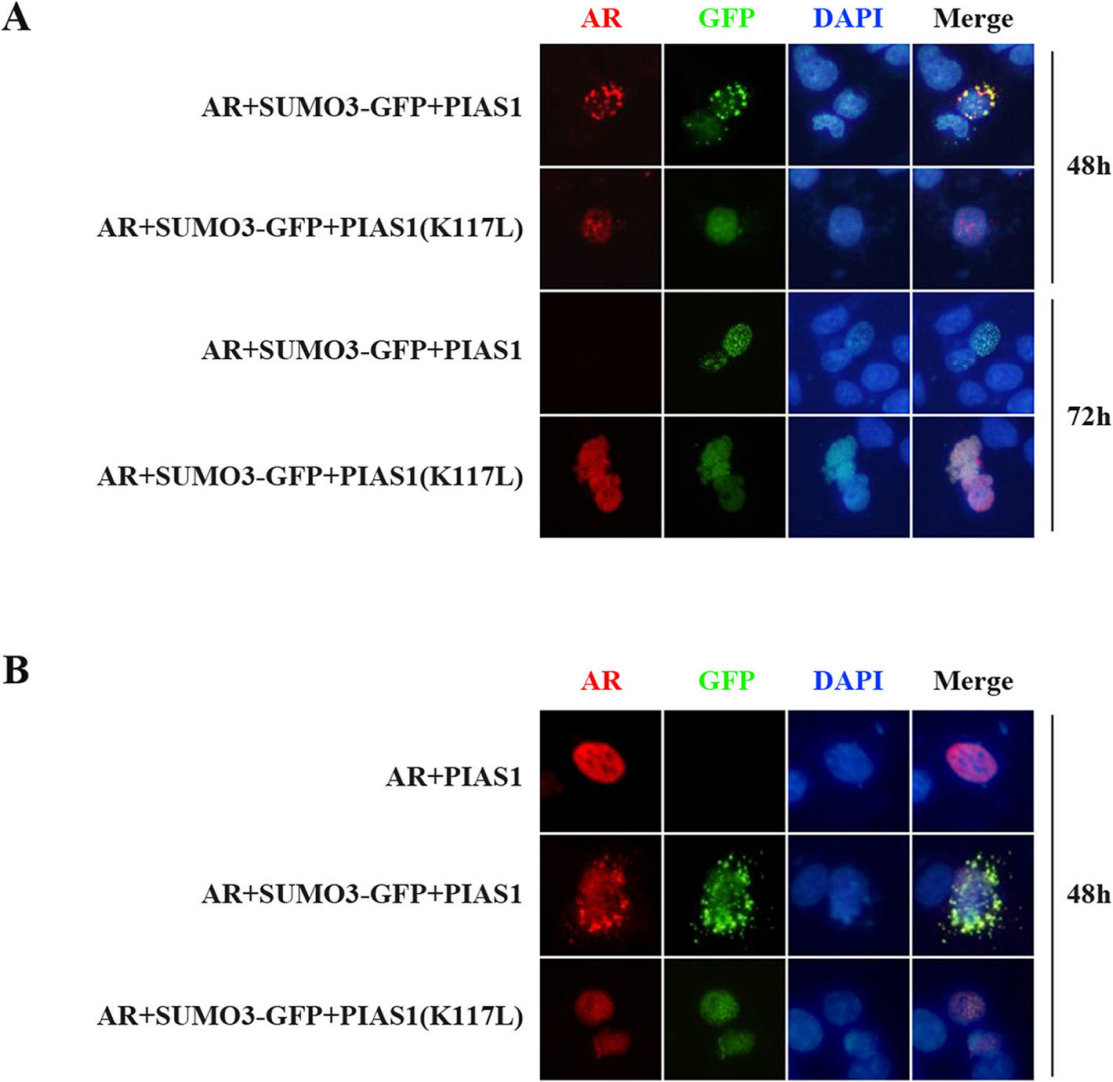
Inability of PIAS1(K117 L) to promote cytoplasmic translocation of AR from nucleus. (**a**) DU145 cells were transiently co-transfected with flag-AR, GFP-SUMO3 and myc-PIAS1 or myc-PIAS1 (K117 L) for 48 h or 72 h. Cells were fixed and stained with anti-flag mouse monoclonal antibody(red). Nuclei were visualized by DAPI staining. Representative images of transfected cells were shown. (**b**) DU145 cells were transiently co-transfected with flag-AR, GFP-SUMO3 and myc-PIAS1 or myc-PIAS1 (K117 L) for 48 h. Cells were fixed and stained with anti-myc mouse monoclonal antibody (red). Nuclei were visualized by DAPI staining. Representative images of transfected cells were shown

To establish the functional relationship between the AR-sumoylated PIAS1 interaction and AR cytoplasmic translocation, we compared ectopic AR localization in cells transfected with PIAS1 or PIAS1 K117 L, along with SUMO3. Consistent with the above results (Fig. 1), wildtype PIAS1 together with SUMO3 can promote partial AR relocation to the cytoplasm. However, sumoylation-deficient PIAS1 K117 L in the presence of SUMO3 cannot promote AR export from the nucleus (Fig. 6a). Furthermore, the PIAS1 K117 L mutant also remains in the nucleus, where wildtype PIAS1 is partially found in the cytoplasm (Fig. 6b). These findings indicate the involvement of sumoylated-PIAS1 in AR cytoplasmic translocation.

**Fig. 7:**
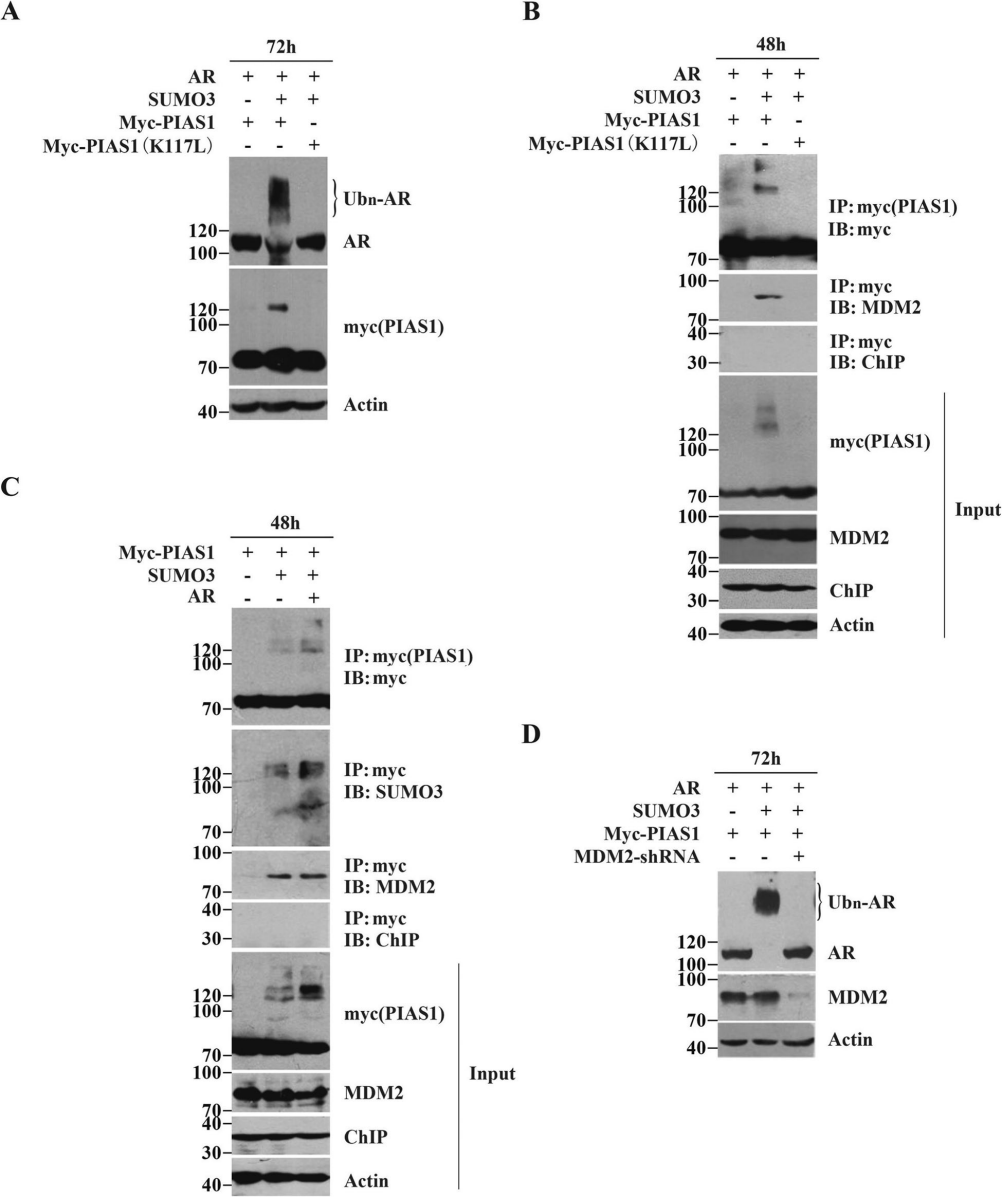
MDM2 is recruited by SUMO3 modificd PIAS1 and required for degradation of AR. (**a**) DU145 cells were transiently co-transfected with flag-AR, GFP-SUMO3 and myc-PIAS1 or myc-PIAS1 (K117 L) for 72 h. Whole-cell lysates were immunoblotted with anti-AR, anti-myc and anti-actin antibodies. (**b**) DU145 cells were transiently co-transfected as described in A for 48 h. Whole-cell lysates were immunoprepapited with anti-myc antibodies. The immunoprepapite was then detected by indicated antibodies against myc, MDM2 and ChIP. Whole-cell lysates (Input) were also immunoblotted with anti-myc, anti-MDM2, anti-ChIP and anti-actin antibodies. (**c**) DU145 cells were transiently co-transfected with PIAS1, empty vector or GFP-SUMO3 or GFP-SUMO3 and myc-PIAS1. Whole-cell lysates were immunoprepapited with anti-myc antibody. The immunoprepapite was then detected by indicated antibodies against myc, SUMO3, MDM2 and ChIP. Whole-cell lysates (Input) were also immunoblotted with anti-myc, anti-MDM2, anti-ChIP and anti-actin antibodies. (**d**) DU145 cells were transiently co-transfected with flag-AR, myc-PIAS1 and empty vector or GFP-SUMO3 or GFP-SUMO3 and MDM2 shRNA for 72 h. Whole-cell lysates were immunoblotted with anti-AR, anti-myc and anti-actin antibodies

Having demonstrated the relevance of PIAS1 sumoylation and AR nuclear export, we next assessed the influence of PIAS1 sumoylation on AR proteolysis. For this, we investigated whether the PIAS1 K117 L mutant could restore AR degradation. As shown in Fig. 7a, elimination of SUMO3-modified K117 in PIAS1 significantly impaired AR ubiquitination and degradation. This finding led us to conclude that sumoylation of PIAS1 itself plays a crucial role in AR ubiquitin-mediated degradation, followed by AR cytoplasmic relocation.

### MDM2 is recruited by SUMO3-modified PIAS1 to induce AR degradation

Proteins are selected for proteasome-mediated degradation by specific ubiquitin E3 ligases that ubiquinate target proteins. Two different E3s (MDM2 and CHIP) are commonly known to generate poly-ubiquitin chains on AR that target it for ubiquitin-proteasome mediated degradation [13, 15, 16]. Because of this, we evaluated whether SUMO3-modified PIAS1 could bind to either of these two E3s. Immunoprecipitation showed that PIAS1 along with SUMO3 in the presence of AR could efficiently co-precipitate with E3 ligase MDM2. Sumoylation-deficient PIAS1 K117 L mutant, however, could not interact with MDM2 (Fig. 7b). Therefore, our results indicate that MDM2 is selectively recruited in cells that co-express PIAS1, SUMO3, and AR.

To confirm direct binding of MDM2 to Sumoylated-PIAS1, we performed immunoprecipitations using the myc antibody in cells expressing PIAS1 and SUMO3 together, with or without AR. As expected, MDM2 is equally co-precipitated in both myc-immunoprecipitations (Fig. 7c IB: MDM2 lane 2 and 3), but not in cells co-transfected with PIAS1 and the vectors (Fig. 7c IB: MDM2 lane 1). Since the additional, slowly migrating PIAS1 band at ~120KD (i.e. representing SUMO3 modification of PIAS1, Fig. 7c IB: SUMO3) is consistent with MDM2 recruitment, we concluded that SUMO3-modified PIAS1 directly binds to MDM2.

As further evidence of the role of MDM2 in SUMO3-modified PIAS1-mediated AR degradation, we used MDM2 siRNA. The expression of endogenous MDM2 was significantly decreased by MDM2 shRNA, which in turn promoted AR expression (Fig. 7d), thus confirming that AR turnover mediated by SUMO3-modified PIAS1 was MDM2-dependent.

### MDM2 is not required for SUMO3-modified PIAS1-induced AR nuclear export

**Fig. 8:**
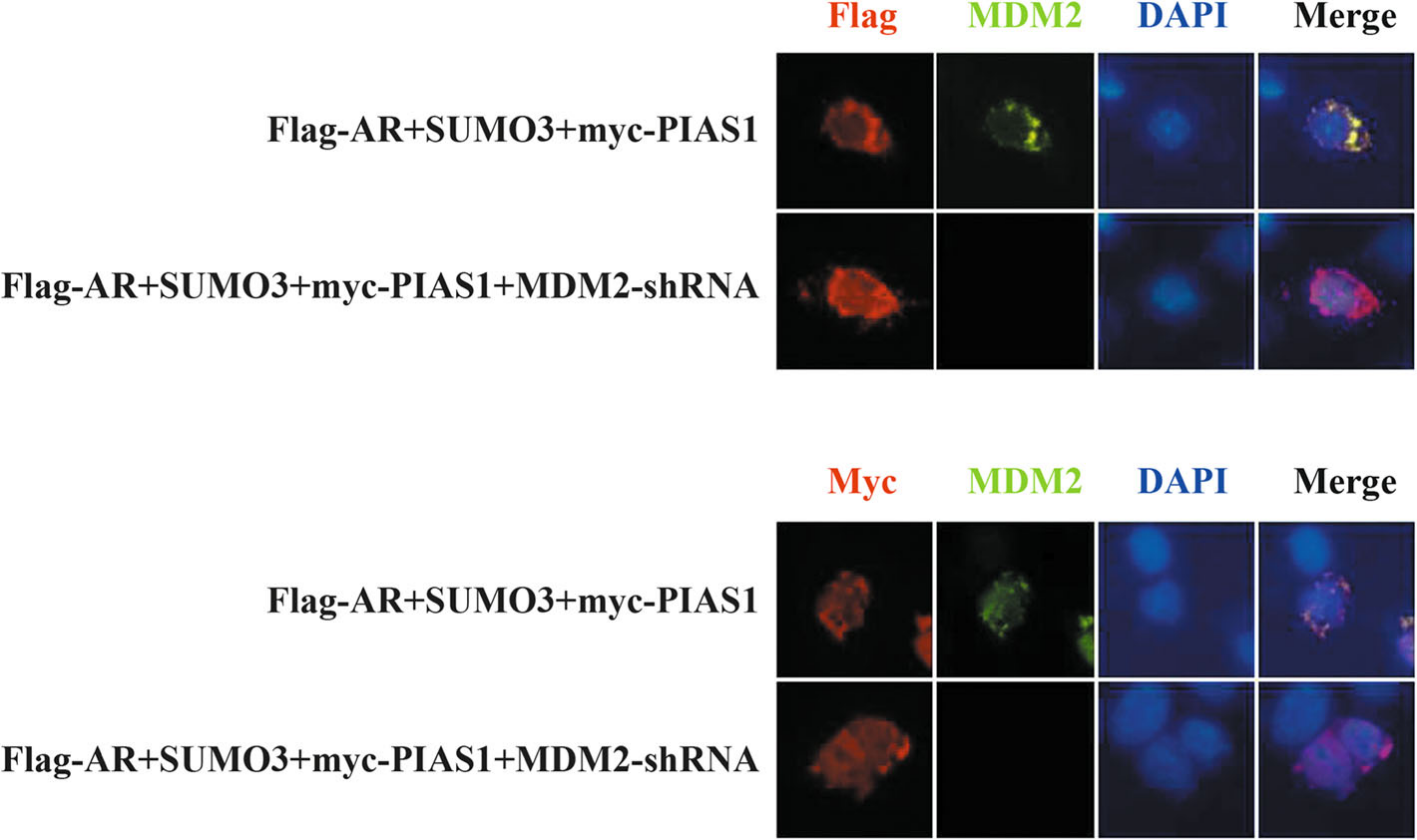
MDM2 is not required for SUMO3 modified PIAS1 induced AR nuclear export. DU145 cells were transiently co-transfected with flag-AR, SUMO3, myc-PIAS1 and control shRNA or MDM2 shRNA for 48 h. Cells were then fixed and stained with anti-MDM2 (green) and anti-flag (red) or anti-myc mouse monoclonal antibody (red). Nuclei were visualized by DAPI staining. Representative images of transfected cells were shown

Finally, we asked whether MDM2 also played a role in cytoplasmic relocation of the SUMO3-PIAS1/AR complex. Here, we found that depletion of endogenous MDM2 had no effect on either AR nuclear export or PIAS1 nuclear export (Fig. 8), suggesting that MDM2 plays no role in cytoplasmic relocation of the SUMO3-PIAS1/AR complex.

## Discussion

AR plays a central role in the carcinogenesis and CRPC transformation in PCa [2, 6]. The up-regulated AR protein level either by the overexpression of AR gene or by the impaired degradation pathway of AR protein, leads to therapy resistant of CRPC [6]. Thus, inhibition of AR signal through increasing the proteasomal-mediated degradation of AR is a promising drug target in CRPC. In the present study, we identified a novel AR degradation associated sumoylation pathway, in which SUMO3 modified AR by PIAS1 (a SUMO E3 ligase), initiated AR cytoplasmic translocation, and further degradation via recruitment of ubiquitin E3 ligase MDM2 (a ubiquitin E3 ligase). Thus, we explored a crosstalk mechanism between AR sumoylation and ubiquitination pathways mediated by a novel PIAS1/SUMO3/AR complex.

Sumoylation can alter Substrate protein function and regulate cellular distribution of conjugated-proteins. In most cases, SUMO modification has been primarily described as impacting on nuclear import of target proteins, such as the first known substrate RanGAP1 [33]. Nevertheless, other studies have shown that SUMOs are involved in nuclear export of modified proteins, such as tumor suppressor TEL [34], Smad3 [35], p53 [36] and proteasome activator REGγ [37]. In our study, SUMO E3 ligase PIAS1, together with SUMO3, markedly increased cytoplasmic trans-localization of AR, which normally stays in the nucleus in PCA cells (Fig. 1). Although increased SUMO3-modified forms of AR by PIAS1/SUMO3 overexpression are detected (Figs. 2c and Fig.3a), only mutation of sumo-acceptor K386, and not mutation of sumo-acceptor K520, impairs nuclear export of AR (Fig. 3b), indicating a novel mechanism for AR nuclear export. This situation is different from regulation of AR-mediated transactivation by SUMO3 or PIASy, which is also independent of SUMO3-conjugation of AR [23, 38]; and different from sumoylation-independent regulation by SUMO machinery, including SUMO and SUMO E3 ligases PIASs, found in many other transcription factors FLI-1 [39] and LEF-1 [40]. Therefore, we hypothesize that SUMO3 modification of AR participates in some unknown regulation process of AR, rather than its cytoplasmic translocation, or that it is an incidental consequence of PIAS1/SUMO3 overexpression accompanying AR re-localization.

AR consists of four distinct domains, an N-terminal domain (NTD), a DNA-binding domain (DBD), a ligand-binding domain (LBD), and a hinge region that separates the LBD from the DBD [4]. Access of AR to the cell nucleus is ensured by two nuclear localization signals (NLSs): NLS1 in the DBD and hinge region, and NLS2 in the LBD [41, 42]. The DBD which contains the partial NLS1, has yet been reported to mediate direct protein-protein interactions with PIAS1 [20]. Our results show the crucial role of sumoylated-PIAS1 in AR nuclear export. However, it appears that there no direct binding of sumoylated-PIAS1 to any NLS sequence in AR to hinder nuclear localization of AR (data not shown). Here, we found that AR K386 and K845 in the NTD and LBD, respectively, contribute to interactions with sumoylated-PIAS1.

**Fig. 9:**
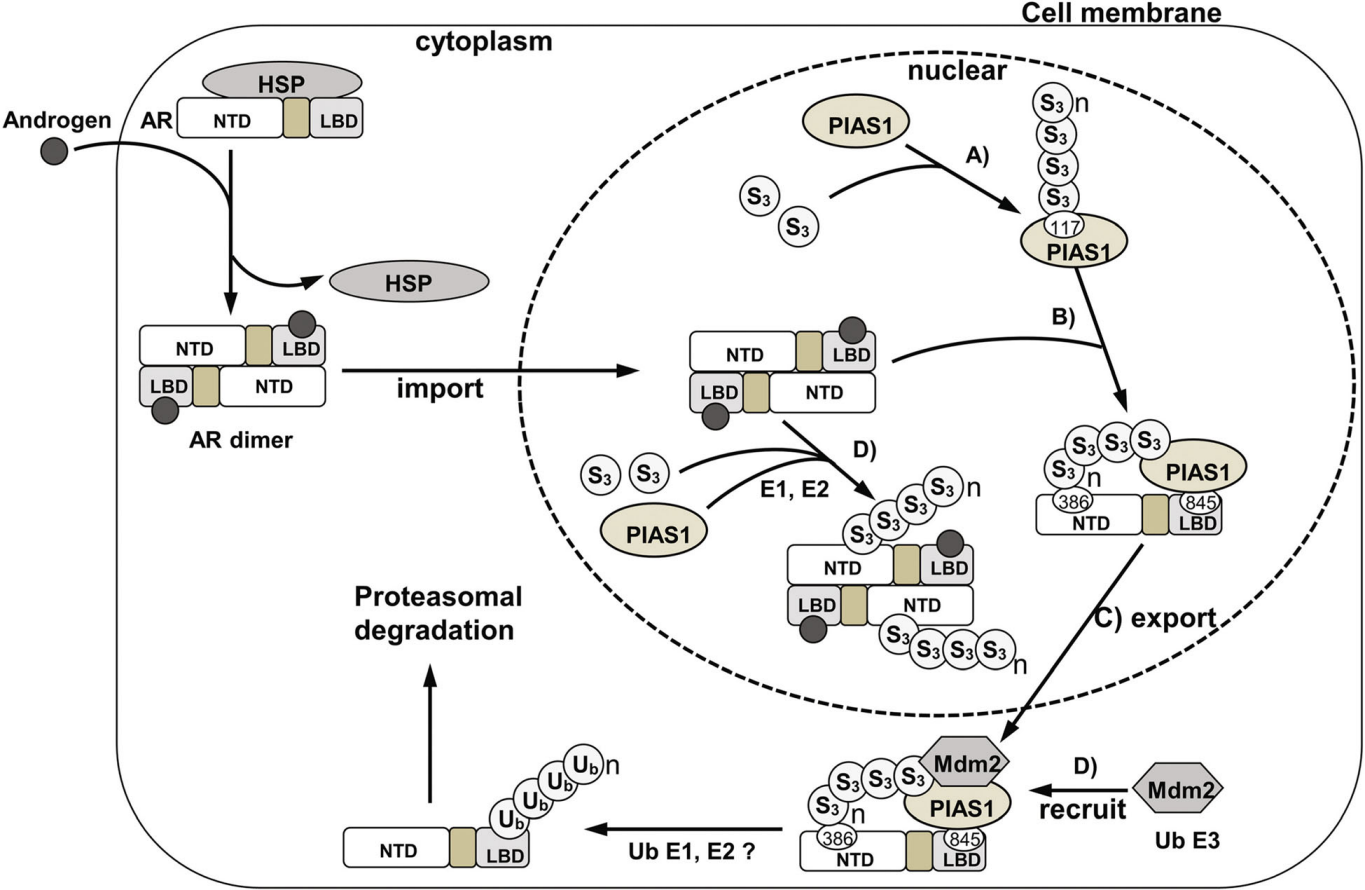
Model for the regulation of AR subcellular localization and turnover by sumoylation and ubiquitination systems. In castration-resistant prostate cancer cells, the binding of androgen contained in the serum makes AR to be released from the cytoplasmic associated heat shock proteins (HSP) and translocate to the nucleus; likewise, the overexpressed PIAS1 and SUMO3 are also gathered in nucleus. SUMO3 can be conjugated to the 117th lysine of PIAS1 which is a SUMO E3 ligase itself (**a**), and then the sumoylated PIAS1 recruit the MDM2 protein(**b**) and also interact with AR through its 386th and 845th lysines, which may block the AR dimer formation (**c**), further resulting in the nuclear export of AR and its binding partners. The MDM2 cooperating with ubiquitin E1 and E2 promotes the polyubiquitination of AR and its subsequent proteasome-mediated degradation. The SUMO3 modification of partial AR is also accompanied in this process (**d**)

Many proteins that are exported from the nucleus, like P53, are degraded in the cytoplasm [25]. Moreover, the nuclear export signal from AR can also be regulated in human prostate cells by ubiquination and proteasome-dependent degradation [24]. The results presented here link the nuclear export of AR caused by sumoylated-PIAS1 to its proteasome-dependent degradation. Sumoylation can influence protein stability by crosstalk with ubiquitination of the same substrate. Because SUMO can competitively conjugate to the same lysine sites on target proteins as ubiquitin, it is well-accepted that sumoylation can stabilize target proteins, as exemplified by PCNA [43], IKBα [44] and Smad4 [45]. More recently, there is increasing evidence that sumoylation can also target some proteins for proteasomal degradation [26, 46], e.g. HIF1-α [47], BMAL1 [48], EGR1 [49], PML and PML-RARA [50]. The model underlying SUMO-dependent ubquitination is provided by the study of HIF-α [47] and PML [50]. This model is based mainly on the interaction of sumoylated substrate with its own particular ubiquitin E3 ligase, which characteristically contains multiple SUMO interaction motifs (SIMs) in the protein sequence. These multiple SIMs-contain ubiquitin E3 ligases that can recognize the SUMO moiety in SUMO conjugates, and thus facilitate their degradation [26, 27]. Nevertheless, since ubiquitin E3 ligases for BMAL1 and EGR-1 and the ubiquitin E3 ligase CK2 for sumoylated PML do not contain SIMs, this model seems not to be the general mechanism of action for all SUMO-dependent ubiquitination [27]. In the present study, we discovered that PIAS1 and SUMO3 co-overexpression leads to proteasomal degradation of AR following its nuclear export (Fig. 2). Based primarily on two observations: (a) effect of AR sumo-acceptor mutations in AR nuclear export, with only K386R preventing ubiquitination-mediated degradation of AR (Fig. 3c) and (b) PIAS1 interacts with AR, but not with SUMO3-modified AR (Fig. 5b), this finding provides a novel aspect to the mechanism of action in SUMO system-mediated substrate degradation. Indeed, sumoylation of SUMO E3 ligase PIAS1 itself is required for AR degradation by the recruitment of AR ubiquitin E3 ligase MDM2 (Fig. 9).

PIAS proteins function widely as common co-transcriptional factors and SUMO E3 ligases. Although self-sumoylation of PIASs had yet to be addressed in vitro and in vivo [21, 28, 29], the biological function of sumoylated PIASs are almost unknown. Only a single study on the role of sumoylated PIASs has been reported. Ihara et al demonstrated that SUMO1 modification of PIASy is necessary for PIASy-dependent activation of tcf-4 [30]. In our study, we discovered that SUMO3-modified PIAS1 can regulate AR cellular distribution and protein stability. In addition, we discovered here that sumoylated PIAS1, can specifically associate with AR ubiquitin E3 ligase MDM2, and enhance interaction of proteins by self-sumoylation of PIASs. To our knowledge, this study is the first report describing the biological role and molecular details of PIAS1 sumoylation.

## Conclusion

### We explore a crosstalk mechanism between AR sumoylation and ubquitination

We explored a crosstalk mechanism between AR sumoylation and ubquitination, which is specifically mediated by PIAS1 and SUMO3. We discovered that SUMO3 modification by PIAS1 modulates AR cellular distribution and stability.

### A novel complex of PIAS1/SUMO3/AR is identified functioning for AR cytoplasmic translocation and degradation

We identified a PIAS1/SUMO3/AR complex, in which SUMO3 modified AR by PIAS1 (a SUMO E3 ligase), which functions for AR cytoplasmic translocation, and further degradation via recruitment of ubiquitin E3 ligase MDM2.

### AR sumoylation site and ubquitination site are characterized for PIAS1/SUMO3/AR translocation complex

Two AR sumoylation sites, K386 and K520, and two AR ubiquitination sites K845and K847R was reported in the previous study. Here we identified AR sumoylation site K386 and ubiquitination site K845, were necessary for the formation and function of PIAS1/SUMO3/AR cytoplasmic translocation complex.

### PlAS1-self sumoylation initiate the formation of PIAS1/SUMO3/AR complex

We demonstrated SUMO3 modification of PlAS1 K117 is necessary for PIAS1/SUMO3 complex formation and itself sumoylation further mediated AR sumoylation (sumo3-conjugated), which induced AR cytoplasmic translocation, and then SUMO3-modified PIAS1 recruited MDM2 to induce AR degradation.

### We provide valuable insight into AR regulation in CRPC

Our findings reveal a previously unknown crosstalk between the sumoylation and ubiquitination in castration-resistant PCa cells, and thus provide valuable insight into AR regulation in CRPC. Therefore, this study may prove useful to developing strategies for therapeutic intervention against AR overexpression in CRPC.

## Material and methods

### Experimental design and Main methods

SUMO1 has only around 45% identity with both SUMO2 and 3 [17]. To access the potential effects of SUMO modification on AR intracellular translocation or on AR degradation in CRPC, AR, each of PIAS [1–4], and each of SUMO [1, 3] plasmids were co-transfected into DU145 cells, a CRPC cell line. AR subcellular localization and degradation in immunofluorescence assays were observed in a total of 96 h. AR mRNA levels were measured in RNA extraction and RT-PCR experiments. AR ubiquitination analysis was carried out by co-immunoprecipitation experiments and confirmed by MG132 (a proteasome inhibitor) treatment. Through above experiments, a novel AR nuclear export/degradation associated complex of PIAS1/SUMO3/AR was identified. Next, we identified AR sumoylation and its sites in the complex by co-immunoprecipitation of AR wildtype vs AR mutants overexpressed cells. Through mammalian two-hybrid assays and luciferase assays, the inner-interactions between each other proteins in the PIAS1/SUMO3/AR complex were verified. Furthermore, we detected the roles of PIAS1 in this complex. Firstly, PIAS1 self-sumoylation and involved residues were determined by the co-immunoprecipitation experiments. Secondly, the effect of PIAS1 self-sumoylation in AR sumoylation was detected in co-immunoprecipitation assays, and PIAS1 self-sumoylation mediated AR nuclear export was observed in immunofluorescence experiments. Finally, the roles of PIAS1 self-sumoylation in MDM2 (a ubiquitin E3 ligase) recognization were determined in the co-immunoprecipitation experiments and the recruitment of MDM2 by PIAS1/SUMO3/AR complex were verified by shRNA knockdown in immunofluorescence experiments. Thus, the importance of PIAS1 in SUMO3 conjugation and in AR sumoylation, nuclear export and degradation was demonstrated in this study.

### Reagents, antibodies and plasmids

Proteasome inhibitors MG132 was purchased from Sigma. The following primary antibodies were used in this study: SUMO3, Ubiquitin and AR rabbit polyclonal antibodies (Santa Cruz), PIAS1 rabbit polyclonal antibody (Cell signaling), MDM2 rabbit polyclonal antibody (Bioss), Flag tag and myc tag mouse monoclonal antibodies (TransGene Biotech). PIAS family genes were subcloned into pCMV-myc vector (Clontech). Full-length AR and various site mutations in pcDNA3-flag vector, including AR (K386R), AR (K520R), AR (K845R), and AR (K847R), were separately subcloned into VP16 vector by NdeI and XbaI. Full-length PIAS1 and a sumoylation site mutation PIAS1 (K117 L) were separately subcloned into GAL4 vector by TthIII and XbaI.Other plasmids have been described in Acknowledgments.

### Site-directed mutagenesis

Site-directed mutagenesis were used to engineer residues K386, K520, K845 and K847 site mutants of AR and residue K117 site mutant of PIAS1. Briefly, primers that included the residue mutation flanked by a wild-type sequence on either side, were generated. A PCR reaction produced a new complete copy of the plasmid containing the mutation coded for by the primers. The remaining parent plasmid pcDNA3-flag-AR or pCMV-myc-PIAS1 in PCR product was then digested by Dpn I and PCR product without parent plasmid was subsequently transformed into DH5αE.coli. DNA sequence containing the point mutation was verified by DNA sequencing.

### RNA extraction and RT-PCR

RNA extraction and reverser transcriptional PCR analysis were used to quantify the change of AR mRNA levels induced by PIAS1 and SUMO3 at different time points. We harvested cultured cells in TRIzol (Invitrogen) and extracted total RNA following the manufacturer’s instructions. Two μg total RNA were transcribed (RT) into 20 μl cDNA by SuperScript III kit (Invitrogen) with oligo (dT) primer. Two μl reverse transcribed cDNA were used for PCR. We designed AR primers for PCR to measure its gene expression level and used the β-actin expression level as control. The AR primers were 5′- CAGAAGACCTGCCTGATCT-3′ (forward) and 5′- CATCCCTGCTTCATAACAT-3′ (reverse). The β-actin primers were 5′- CATCCTGCGTCTGGACCTG-3′ (forward) and 5′- ATCTCCTTCTGCATCCTGTC-3′ (reverse).

### Cell culture, transient transfections

The human prostate cancer cell line DU145 was cultured in DMEM medium (GIBCO) plus 5% FBS. Transient transfections were carried out using Lipofectamine 2000™ (Invitrogen) following the manufacturer’s protocol.

### Mammalian two-hybrid assays and luciferase assays

Mammalian two-hybrid assays and luciferase assays were employed to determine the protein-protein interactions between wild type AR or its ubiquitination site (K845R, K847R) mutants or its sumoylation site (K386R, K520R) mutants, and wildtype PIAS1 or its sumoylation site K117 L mutant, with or without SUMO3. Cells were transfected overnight at ~ 80% confluence in 48-well plates with 0.1 μg of 5 × GAL4Luc3 reporter and other expression vectors as indicated in the figures and legends. Renilla luciferase reporter was used as an internal control. After 48 h transfection, firefly luciferase activities were measured by using the Dual-Luciferase Reporter Assay System (Promega) and the ratio of firefly luciferase activity to Renilla luciferase activity was calculated as Relative luciferase activity. The results reflect the mean and standard deviation from triplicate samples.

### Immunofluorescence

Immunofluorescent staining assays were performed to assess nuclear export of AR, sub-cellular localization of PIASs, SUMO1/3, MDM2 and molecular co-localizations. After transfection, cells were fixed with 3% formaldehyde for 30 min and then permeabilized with 0.1% Triton-100/PBS for 10 min. After being pre-blocked with 1% BSA/PBS, samples were incubated with indicated primary antibodies for 2 h, followed by incubation with fluorophore-conjugated secondary antibodies (Proteintech Group). Nuclei were counter-stained with 4′,6- diamidino-2-phenylindole (DAPI) after secondary antibody incubation. Cells were examined by fluorescence microscopy (Olympus).

### Immunoprecipitation and Immunoblot analysis

Immunoprecipitation and immunoblots were used to assess sumoylation of AR catalyzed by PIAS1, PIAS1 self-sumoylation, ubiquitination/degradation of AR and its protein levels, the inner complex formation of SUMO3 modified PIAS1 and AR, and also the binding of MDM2 to SUMO3 modified PIAS1. DU145 cells were transfected with various vectors for proper time and cross-linked with 1-2 mM dithio-bis succinimidyl propionate for 30 min. To prepare a total cell extract, transfected cells were lysed in lysis buffer (50 mM Tris-HCl, pH 7.4/150 mM NaCl/1% NP-40/1 mM EDTA) supplemented with the Complete Protease Inhibitor Mixture (Roche). Pre-cleared lysates were then incubated with pre-equilibrated protein-A or protein-G-Sepharose beads with either AR polyclonal antibody (Santa Cruz) or myc tag monoclonal antibody (TransGen Biotech) at 4 °C for 3 h. Eluted proteins were analyzed by immunoblots using SUMO3, ubiquitin, PIAS1 and AR antibodies at the appropriate dilutions.

### Statistical analysis

All results shown are reported as the mean ± standard deviation (SD). The One-way Anova was used to compare the means of 2 independent groups in more than two groups. Statistical significance was assumed for a tailed *p* < 0.05 or *p* < 0.01 using SPSS17.0.

### Availability

GPS-SUMO is an open software freely available for academic research at website: http://sumosp.biocuckoo.org/download.php

## Data Availability

The data sets used and/or analyzed during the current study are available from the corresponding author on reasonable request.

## Abbreviations

AR: Androgen receptor
CRPC: Castration-resistant prostate cancer
DAPI: 4′,6- diamidino-2-phenylindole
DBD: DNA-binding domain
LBD: Ligand-binding domain
MDM2: Double minute 2 protein
NLS: Nuclear localization signals
NTD: N-terminal domain
PCa: Prostate cancer
PIAS: Protein inhibitor of activated STAT
RT-PCR: Reverse Transcriptase-Polymerase Chain Reaction
SIMs: SUMO interaction motifs
SUMO: Small ubiquitin-related modifier
